# Bio-Augmentation of *Cupriavidus* sp. CY-1 into 2,4-D Contaminated Soil: Microbial Community Analysis by Culture Dependent and Independent Techniques

**DOI:** 10.1371/journal.pone.0145057

**Published:** 2015-12-28

**Authors:** Young-Cheol Chang, M. Venkateswar Reddy, Honoka Umemoto, Yuki Sato, Mi-Hye Kang, Yuka Yajima, Shintaro Kikuchi

**Affiliations:** 1 Course of Chemical and Biological Engineering, Division of Sustainable and Environmental Engineering, College of Environmental Technology, Muroran Institute of Technology, 27–1 Mizumoto, Muroran, 050–8585, Japan; 2 Seoul Metropolitan Government Research Institute of Public Health and Environment, 202–3 Yangjae-dong, Seocho-gu, Seoul, 137–893, Republic of Korea; 3 Graduate School of Medicine, Kyoto University, Yoshidakonoe-cho, Sakyo-ku, Kyoto-shi, Kyoto, 606–8501, Japan; University of Coimbra, PORTUGAL

## Abstract

In the present study, a 2,4-dichlorophenoxyacetic acid (2,4-D) degrading bacterial strain CY-1 was isolated from the forest soil. Based on physiological, biochemical and 16S rRNA gene sequence analysis it was identified as *Cupriavidus* sp. CY-1. Further 2,4-D degradation experiments at different concentrations (200 to 800 mg l^-1^) were carried out using CY-1. Effect of NaCl and KNO_3_ on 2,4-D degradation was also evaluated. Degradation of 2,4-D and the metabolites produced during degradation process were analyzed using high pressure liquid chromatography (HPLC) and GC-MS respectively. The amount of chloride ions produced during the 2,4-D degradation were analyzed by Ion chromatography (IC) and it is stoichiometric with 2,4-D dechlorination. Furthermore two different types of soils collected from two different sources were used for 2,4-D degradation studies. The isolated strain CY-1 was bio-augmented into 2,4-D contaminated soils to analyze its degradation ability. Culture independent methods like denaturing gradient gel electrophoresis (DGGE) and terminal restriction fragment length polymorphism (T-RFLP), and culture dependent methods like colony forming units (CFU) and most probable number (MPN) were used to analyze the survivability of strain CY-1 in contaminated soil. Results of T-RFLP were coincident with the DGGE analysis. From the DGGE, T-RFLP, MPN and HPLC results it was concluded that strain CY-1 effectively degraded 2,4-D without disturbing the ecosystem of soil indigenous microorganisms.

## Introduction

The herbicide 2,4-dichlorophenoxyacetic acid (2,4-D) has been broadly used for the control of broadleaf weeds since its introduction in the 1940s. Different bacteria which degrades 2,4-D have been isolated by many researchers [1] and one of the most comprehensively explored bacteria is *Cupriavidus necator* JMP134 (formerly known as *Alcaligenes eutropha*, *Ralstonia eutropha* and *Wautersia eutropha*) [2]. The pathway and genes accountable for degradation of 2,4-D have been characterized mainly by the use of *C*. *necator* strain JMP134 [1]. Although, a successive 2,4-D contaminated soil remediation has been reported by Manzano et al. [3], it remained unknown whether *Cupriavidus* genus play important role for 2,4-D bioremediation in soil microcosm. Bacterial strain *C*. *necator* JMP134 was isolated from 2,4-D treated agricultural soils and most of the 2,4-D degrading bacteria were isolated from human-disturbed sites [4]. Therefore, there is lack of information about 2,4-D degradation using bacteria isolated from uncontaminated site, and its application in bio-augmentation.

On the other hand, 2,4-D has been using as a herbicide in Japan and the number of 2,4-D degrading strains such as *Bradyrhizobium* sp. strain RD5-C2, *α-*, *β-*, *γ-proteobacteria*, *Burkholderia* sp. *Cupriavidus* sp. and *Sphingobium* sp. have been reported from activated sludge and typical paddy field in geographically different areas in Japan [1, 5–7]. All these reports indicated that microorganisms capable of degrading 2,4-D may exist widely in Japan. However, the application of those strains into the 2,4-D-contaminated soil was not studied in detail. Therefore, further investigation is required. Eventually, a study conducted with strains isolated from unspoiled soils will extend our understanding of the *in situ* remediation strategies in 2,4-D contaminated soil or contaminated with newly introduced toxic organic compounds.

Even though information available about the effects of 2,4-D on soil microbial communities [8–11], complete understanding of the microbial residents related with 2,4-D degradation in soil was problematic due to the boundaries of methods used to recognize and depict populations. Classical cultivation methods, such as most probable number (MPN) and plating were valuable for gaining pure cultures, but the precincts of cultivation methods are broadly renowned. So, culture independent techniques based on phylogenetic and mobile genetic elements (MGEs) are significant for empathy of inhabitants and pathways involved during 2,4-D degradation in soils [2, 12–15]. However, earlier studies have shown that preferences associated with molecular methods [4, 16, 17], as well as cultivability of various 2,4-D degrading bacteria [18] can incline analysis of phenotype profusion and the structure of the original bacterial community.

Macur et al. [19] reported that use of combined techniques (culture dependent and independent) provides accurate assessment of microbial diversity in 2,4-D contaminated soil. Thus, we still need to collect more data relating culture dependent and independent methods in order to formulate accurate bioremediation strategies. The aim of this study was to characterize the 2,4-D degrading strain *Cupriavidus* sp. CY-1 which was isolated from a pristine site located in Muroran, Hokkaido. Identification of 2,4-D degradation potential of CY-1 and optimization of environmental factors such as pH and temperature for better degradation of 2,4-D. Effect of NaCl and KNO_3_ on growth and 2,4-D degradation was also evaluated. Degradation of 2,4-D and the metabolites produced during degradation process were analyzed using high pressure liquid chromatography (HPLC) and gas chromatography-mass spectroscopy (GC-MS) respectively. Amount of chloride ions produced during the 2,4-D degradation were analyzed by Ion chromatography (IC). Further, strain CY-1 was bio-augmented into 2,4-D contaminated soils to analyze its efficiency for degradation of 2,4-D. Culture dependent and independent methods were used to identify the native microbial populations present in soil and existence capacity of strain CY-1 in 2,4-D contaminated soil. Denaturing gradient gel electrophoresis (DGGE) and terminal restriction fragment length polymorphism (T-RFLP) were used as culture independent methods, colony forming unit (CFU) and most probable number (MPN) were used as culture dependent methods.

## Materials and Methods

### Isolation and identification of 2,4-D degrading bacteria

Soil with no history of herbicide contamination collected from the forest located in Muroran city, Hokkaido, Japan were used as inoculum source for isolation of 2,4-D degrading bacteria. Muroran city is authorised and issued the permission for soil collected from forest. Bacteria were isolated using enrichment culture method. For the enrichment of 2,4-D degrading bacteria mineral salt (MS) medium was used. The MS medium contained 1.0 g (NH_4_)_2_SO_4_, 1.0 g K_2_HPO_4_, 0.2 g NaH_2_PO_4_, 0.2 g MgSO_4_ 7H_2_O, 0.05 g NaCl, 0.05 g CaCl_2_, 8.3 mg FeCl_3_.6H_2_O, 1.4 mg MnCl_2_.4H_2_O, 1.17 mg Na_2_MoO_4_ 2H_2_O and 1 mg ZnCl_2_ per one liter of deionized water.

Five grams of soil and 100 ml of autoclaved MS medium supplemented with 100 mg l^-1^ concentration of 2,4-D as the sole carbon source was used as enrichment medium. Flasks were incubated for two months under dark condition at 28°C with agitation speed of 110 rpm. Within two months, sub-culturing was done for 4 times with the time interval of every 2-weeks using the MS medium with similar concentration of 2,4-D. After the enrichment, a 2,4-D utilizing bacterium was successfully isolated using the traditional serial dilution method. To isolate colonies 10-fold diluted enrichment culture was spread on solid agar media which was prepared by MS medium contains 100 mg l^-1^ of 2,4-D, 1.0 g l^-1^ of polypeptone, 0.5 g l^-1^ yeast extract and 2.0% agar. The plates were incubated under aerobic conditions. The procedure was repeated twice to assure pure culture. The purity of isolated culture was confirmed by an inverted microscope (Diaphot TMD300, Nikon, Tokyo, Japan) equipped for simultaneous recording of cell length.

Total genomic DNA was extracted from the isolated colonies using Takara, Nucleospin kit. The 16S rRNA gene fragment was amplified by polymerase chain reaction (PCR) with a pair of universal primers 27 F and 1392 R under standard conditions. DNA sequencing and phylogenetic analysis was performed according to the previously reported method [20]. The physiological characteristics of the isolates were also determined using commercially available identification systems (API 20 E and API 20 NE; bioMérieux, Japan). The 16S rRNA gene sequence was deposited in the DDBJ under accession no: AB604176.

### Growth curve and 2,4-D degradation studies with CY-1

Among the isolated strains, one strain exhibited highest 2,4-D utilization/degradation activity, so it was selected for further experiments. Initially, bacteria was cultivated aerobically for 16 h in MS medium at 28°C by supplementing with 2,4-D at 100 mg l^-1^ concentration as the sole carbon and energy source. After 16 h the culture was harvested by centrifugation (8,000 × g, 5 min, 4°C) and washed twice with 50 mM potassium phosphate buffer (pH 7.0). For growth curve studies, 200 μL of cell suspension was inoculated in separate flasks containing MS medium with different concentrations (200, 300, 500, 600, 700 and 800 mg l^-1^) of 2,4-D. Flasks were kept in rotary shaker at 110 rpm under the aerobic dark condition at 28°C for 120 h. Samples were collected at different time intervals to measure the growth and 2,4-D degradation. Growth was monitored spectrometrically by measuring the absorbance at 600 nm by using UV-spectrometer (UV-1800, Shimadzu, Japan). Degradation of 2,4-D was measured by HPLC. HPLC samples were acidified with phosphoric acid (10%, w/v) to stop the biological reaction, shaken for 2 h with an equal volume of 1:1 (v/v) methanol:ultrapure water and centrifuged at 8,000 × g for 10 min. The organic layer was filtered and then analyzed directly by HPLC. The HPLC analysis was conducted using a Shimadzu HPLC system with an SPD-10AV UV/Vis detector and a Shim-pack VP-ODS column (4.5 × 150 mm, particle size 5 μm; Shimadzu, Kyoto, Japan). In the HPLC analysis, 4:1 (v/v) acetonitrile: 1% acetic acid solution was used as the mobile phase and the detection wavelength was 280 nm. All experiments were performed in triplicates.

### Effect of pH, temperature, NaCl and KNO_3_ on 2,4-D degradation

Bacterial cells were harvested by centrifugation (10,000 × g, 4°C, 15 min) and pellet (0.65 g wet weight) was resuspended in 6 ml of 50 mM Tris-HCl (pH 7.5) containing 5% glycerol and lysed in an ice using a Branson ultrasonic disrupter (Sonifer 250, Danbury, CT, USA) at 30 s flash for 5 min. Centrifugation was done at 19,000 × g and supernatant was collected. Cell debris and unbroken cells were collected and sonicated further with similar conditions. Both supernatants were pooled and filtered through 0.22 μm filter (Adventec, Dismic-25AS, Bedford, USA). The filtrate was served as the enzyme extract. Enzyme extract was reacted at 10°C, 15°C, 20°C, 25°C, 30°C, 35°C and 40°C. To evaluate the effect of pH on 2,4-D degradation, the enzyme extracts were prepared with ultrapure water (pH adjusted to 6.0–9.0), phosphate buffer (pH 6.0–8.0) or Tris-HCl buffer (pH 8.0–9.0). Protein was quantified by the Bradford method using the Bio-Rad protein assay reagent (Bio-Rad, Hercules, California, USA).

To investigate the influence of NaCl and nitrates concentrations on the growth and 2,4-D degradation capacity of the strain CY-1, bacteria were initially grown in MS medium supplemented with 2,4-D at 100 mg l^-1^ concentration up to the mid-log phase. Then 200 μl of cell suspension was inoculated into the fresh MS medium to obtain an optical density of 0.02 at O.D._600_ with 2,4-D at 200 mg l^-1^ concentration. NaCl at different concentrations (0.05, 1, 5, 10, 20, 35 g l^-1^) were added to analyze the influence of NaCl on 2,4-D degradation. After that to analyze the influence of KNO_3_ on 2,4-D degradation, KNO_3_ at different concentrations (0, 10, 100, 500, 1000 mg l^-1^) were added. Control experiments were carried out without adding NaCl and KNO_3_. The flasks were incubated under shaking at 110 rpm for 96 h when cells were grown with NaCl, or during 48 h when grown with KNO_3_ at 30°C temperature.

### Determination of chloride ions and 2,4-D metabolites

Release of chloride ions during 2,4-D degradation were determined. The assay mixture contains: 1 ml of the supernatant, 3 ml of 50 mM Tris-HCl buffer (pH 7.5) and 2,4-D at 221 mg l^-1^ (1 mM). Assay mixture was incubated at room temperature for 5 minutes. The reaction was terminated by adding 0.2 ml of 5 M H_2_SO_4_. The concentration of chloride ions released during 2,4-D degradation were measured by an ionic chromatograph Dionex ICS-1000 equipped with a conductivity detector (Dionex Co., CA, USA), using a 4 mm anionic exchanger column, IonPack AS9-HC. The injection volume was 25 μl and mobile phase was a 9 mM sodium carbonate solution with flow rate of 1 ml/min.

The metabolites produced during 2,4-D degradation were determined by GC-MS. The samples collected at different time intervals were shaken for 3 min with an equal volume of 1:1 (v/v) ethyl acetate and centrifuged at 8,000 × g for 10 min. The organic layer was then extracted and analyzed directly by GC-MS. Prior to the GC-MS analysis, the extract was dried under a nitrogen flow and derived by trimethylsilylation (TMS) using a BSTFA-acetonitrile solution at 60°C for 1 h. The GC-MS analysis was conducted on a Shimadzu GC-MS system (GCMS-QP2010) with an Rxi-5ms capillary column (30 m, 0.25 mm ID, 1.00 lm df; Restek, Pennsylvania, USA) or a PTE-5 (30 m, 0.25 mm ID; Supelco Inc., Pennsylvania, USA).

### Degradation of 2,4-D using soil bacteria

**Fig 1:**
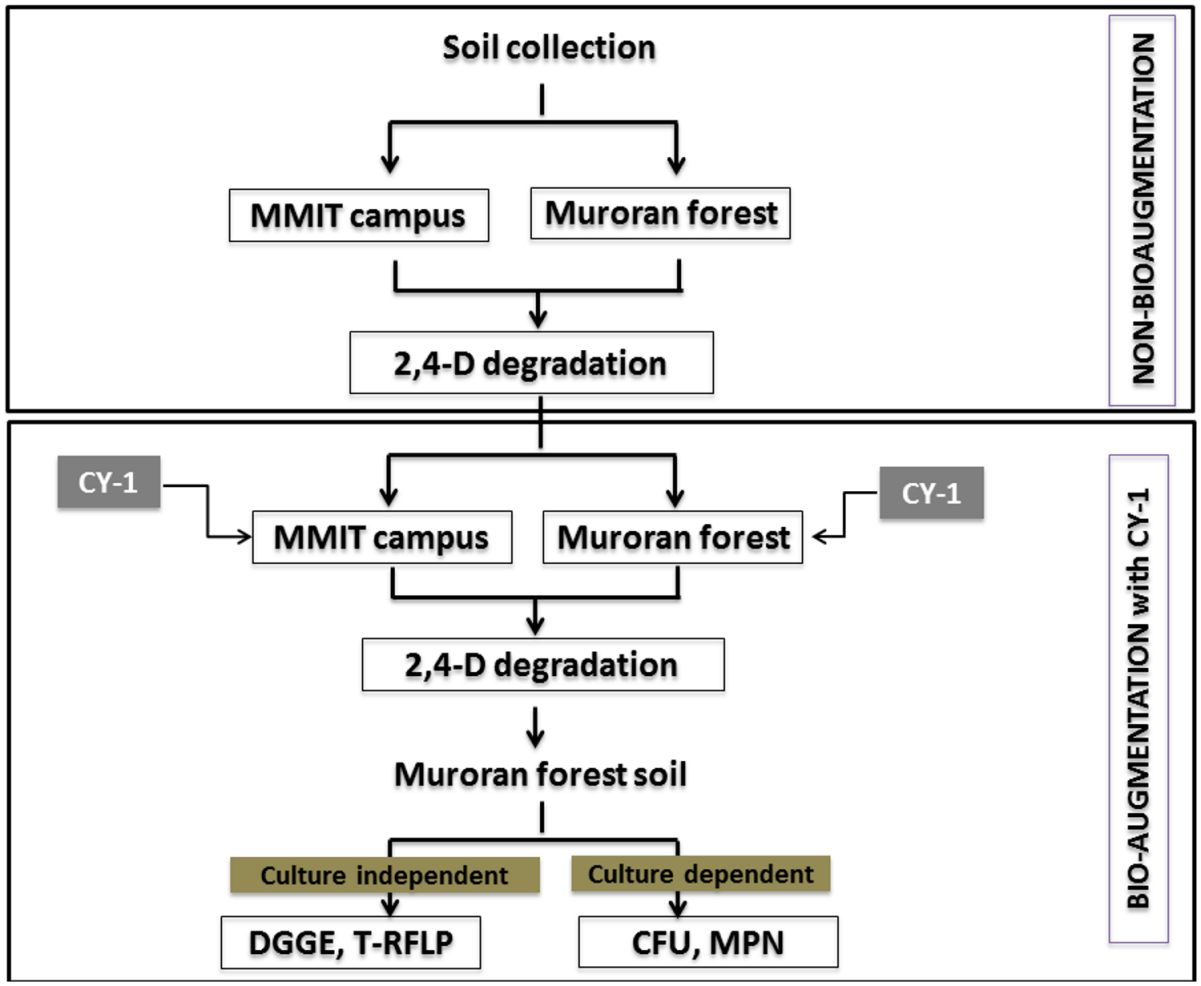
Schematic diagram of experimental methodology.

Two different types of soils *i*.*e*., soil collected from Muroran Institute of Technology (MMIT) campus and soil collected from forest were used for 2,4-D degradation. Muroran city is authorised and issued the permission for soil collected from forest and the president of MMIT is authorised and issued the permission for soil collected from MMIT campus. Soil samples were taken at 0–20 cm depth. Table 1 shows the characteristics of both soils used in the study. Two types of experiments were carried out *i*.*e*., non-bioaugmentation and bio-augmentation (Fig 1). For non-bioaugmentation experiments only soils were used, but for bio-augmentation experiments, 4 ml of CY-1 culture solution (as initial cell number; 2.0×10^7^~1.0×10^8^ colony-forming units (cfu)/ml) was inoculated into soils. For both non-bioaugmentation and bio-augmentation experiments soils were contaminated with 100 mg/kg of 2,4-D. After proper mixing of 5 g of soil with 4 ml of water in 30-ml vials, 2,4-D was added separately. Experiments were carried out for 7 days at static conditions. Samples were collected at different time intervals *i*.*e*., 0 day, 1 day, 3 day and 7 day for HPLC and microbial diversity analysis.

**Table 1:** Physico-chemical characteristics of soil.

Property	Campus soil	Forest soil
Clay (%)	33	39
Silt (%)	31	30
Sand (%)	36	31
Carbon [g·C/l]	21.2	35.1
TOC [%(w/v)]	0.21	0.34
Moisture	4.1	5.2
pH	5.2	5.7
Nitrate [mg l^-1^]	33	21

### Soil microbial diversity analysis

#### CFU and MPN

In order to estimate the culturable aerobic heterotrophic bacteria, 5 g of conditioned forest soil with 10 ml of sodium tri phosphate solution (5 mg l^-1^) was added in glass bottles. After 10 minutes shaking at room temperature, 1 ml of supernatant added into 9 ml of fresh sodium tri phosphate solution (10^−1^ dilution). Dilutions were made from 10^−1^ to 10^−5^. Aliquots (0.1 ml) of samples were streaked onto 1/10 strength nutrient agar (Eiken Chemical Co., Ltd., Tokyo, Japan) plates. Plates were incubated for 2 days at 3000b0030C and the visible colonies were counted. MPN was done to calculate the culturable 2,4-D degrading bacteria (MPN_2,4-D_) present in forest soil samples. Soil samples at different time intervals were used for MPN. Soil samples in each vial were suspended in 0.85% NaCl and made 10-fold dilutions to estimate MPN_2,4-D_. Aliquots (1.0 ml) of appropriate dilutions were transferred to 10 ml of MS medium (5 replicates per dilution) containing 100 mg l^-1^ 2,4-D as a sole carbon source. Plates were incubated for 7 days at 30°C temperature. Vials were scored positive for 2,4-D degradation when at least 20% depletion of 2,4-D was observed by HPLC analysis. MPN_2,4-D_ were calculated using MPN tables [21]. All experiments were performed in triplicates.

#### DGGE and T-RFLP

DGGE was performed to have information on the diversity of the microbial community of the soil. DNA extraction was done according to Venkateswar Reddy et al. [22]. 16S rRNA gene amplification and DGGE analysis were done as described by Martínez-Alonso et al. [23]. 16S rRNA gene sequencing reactions were performed using the Big Dye Terminator v3.1 cycle sequencing kit (Applied Biosystems). Reactions were run in an Applied Biosystems 3130/3130XL genetic analyzers. Sequences were associated to those deposited in the GenBank nucleotide database using the BLAST program and were aligned with the closest matches using the CLUSTALW function of MEGA [24]. The same soil samples which used for DGGE analysis were used for T-RFLP analysis. For T-RFLP analysis, 10 g of soil was collected at different time intervals, total genomic DNA was extracted and purified from each soil sample using phenol-chloroform method [22]. Bacterial 16S rDNA was amplified using PCR with the universal primers, 27 F labelled with 6-carboxy-fluorescein (6-FAM) and 1492 R. T-RFLP analysis was accompanied according to the method described by Dunbar et al. [25].

## Results and Discussion

### Biochemical characterization of strain CY-1

The isolated strain CY-1 is 2,4-D utilizing, aerobic, Gram-negative and rod-shaped bacterium. Results of physiological and biochemical tests of CY-1 were presented in S1 Table. The strain was able to produce urease and cytochrome oxidase but not indole or β-glucosidase (S1 Table). Based on these characteristics as well as carbon utilization test, we considered CY-1 belongs to the genus *Cupriavidus*. On the other hand, the 16S rRNA gene sequence and phylogenetic analysis also showed that strain CY-1 was closely related to *Cupriavidus oxalaticus* AB680453 (99% sequence identity) (S1 Fig). Furthermore, the 16S rDNA sequence data revealed that strain CY-1 was very similar to *Ralstonia* sp. LCH1 AB088545 (96% sequence identity) among the known 2,4-D-degrading bacteria [1, 3].

### 2,4-D degradation using CY-1

**Fig 2:**
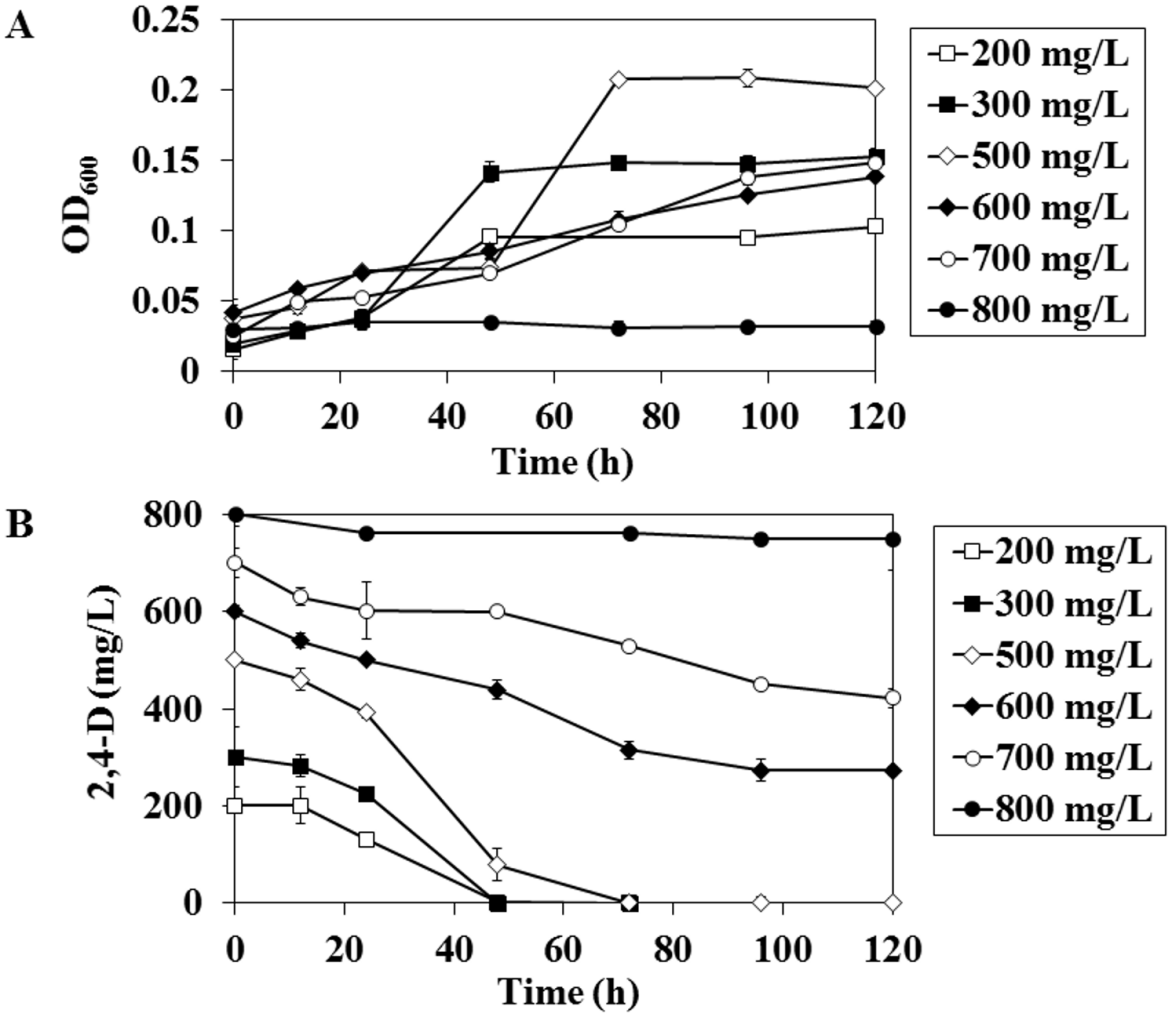
(A) Growth curve of the isolated strain *Cupriavidus* sp. CY-1 with 2,4-D as carbon source; (B) Degradation of 2,4-D using isolated strain *Cupriavidus* sp. CY-1. Bacteria were incubated with 2,4-D at different concentrations (200 to 800 mg l^-1^) as carbon source in MS media at 28°C. Samples were collected at different time intervals and growth was monitored spectrometrically by measuring the absorbance at 600 nm using UV-spectrometer and residual concentration of 2,4-D in the medium were analyzed by HPLC.

2,4-D was completely degraded within 48 h when strain CY-1 was incubated with 200 and 300 mg l^-1^, but for degradation of 500 mg l^-1^ concentration it took 72 h (Fig 2B). CY-1 incubated at 600 and 700 mg l^-1^ showed good growth, the OD values at 120 h were 0.1382 and 0.1485 respectively (Fig 2A), but complete degradation of 2,4-D was not observed up to 120 h. CY-1 incubated at 600 mg l^-1^ and 700 mg l^-1^ showed 55% and 40% removal of 2,4-D respectively within 120 h. At 800 mg l^-1^ concentration, CY-1 showed poor growth and 2,4-D degradation (6%) within 120 h. Strain CY-1 incubated at 300 mg l^-1^ and 500 mg l^-1^ concentration showed faster cell growth, compared with 600 mg l^-1^ and 700 mg l^-1^ concentration. The cell growth was ceased at 800 mg l^-1^ of 2,4-D concentration. These results indicated that strain CY-1 was able to degrade 2,4-D completely up to 500 mg l^-1^ concentration, moderately up to 700 mg l^-1^ concentration. But the strain CY-1 was unable to degrade 2,4-D at 800 mg l^-1^ concentration. It could be assumed that toxicity caused by high concentrations of 2,4-D inhibited the CY-1 growth. Many authors reported that various bacteria were able to grow and utilize 2,4-D as sole carbon source. Three strains of the genus Cupriavidus, stain *C*. *campinensis* BJ71, *C*. HH05 and QH05 were able to degrade 2,4-D (at 500 mg l^-1^ concentration) at 100%, 85% and 90% respectively within 168 h [26]. In our study strain CY-1 took only 72 h for complete removal of 2,4-D at 500 mg l^-1^ concentration. The strain *Bacillus sp*. BJ02 was reported to degrade 100% of 500 mg l^-1^ of 2,4-D in 168 hours [26] and the strain *Pseudomonas cepacia* was reported to degrade 100% of 221 mg l^-1^ of 2,4-D in 51 h [27]. In our study the stain CY-1 took only 48 h for 100% removal of 2,4-D up to 300 mg l^-1^ concentration. In addition, *Azotobacter* SSB81 was able to utilize 1000 mg l^-1^ concentration of 2,4-D as sole carbon source and the growth of SSB81 was primarily inhibited by the presence of 2,4-D in the medium, but after 48 h growth rate was increased slowly [28]. In our study, strain CY-1 grew in 2,4-D in concentrations up to 700 mg l^-1^, beyond this concentration (at 800 mg l^-1^) bacteria were unable to show growth up to 120 h. Bacteria belongs to *Cupriavidus* genus can produce polyhydroxybutyrate (PHB) [29, 30], in addition to degradation of toxic compounds like 2,4-D. PHB is a type of biopolymer produced by many species of bacteria [31, 32]. De Morais et al., [29] investigated the nanofibers production using PHB extracted from *Spirulina* and the bacteria *Cupriavidus necator*.

#### Neutral pH and moderate temperature promotes 2,4-D degradation

The cell extracts of strain CY-1 showed 2,4-D degradation activity at broad pH range of 5–10, with an optimal pH of 7.5 (S2A Fig). At pH 5 and pH 10, degradation activity of CY-1 was 88% and 33% respectively low compared with pH 7.5 respectively. The cell extracts of strain CY-1 showed 2,4-D degradation activity at temperature range of 10–40°C, with an optimal temperature of approximately 30°C (S2B Fig). However, CY-1 showed lower activity (almost 80%) at 10°C. Greer et al. [27] reported that degradation of 2,4-D above pH 7 by *Pseudomonas cepacia* was slow, and not observed at acidic pH (pH, 3.3) or alkaline pH (pH, 8.1). These results were according to our findings, given that CY-1 degradation of 2,4-D was higher at neutral pH (pH 7.5). The results of Greer et al. [27] were similar to our results except at neutral conditions where CY-1 showed higher degradation. Celis et al. [33] operated the aerobic SBR at an ambient temperature (22±2°C) and the anaerobic SBR at lower mesophilic range (30±2°C) by seeding with sludge for degradation of 2,4-D. They reported, aerobic reactor achieved complete 2,4-D utilization at feed concentrations up to 500 mg l^-1^. On the other hand, the anaerobic SBR was able to degrade 120 mg l^-1^ of 2,4-D, which corresponds to 40% of the maximum feed concentration applied [33]. The results of Celis et al. were also consistent with our results, strain CY-1 showed highest 2,4-D degradation approximately at 30°C temperature. Roane et al. [34] also conducted experiments on 2,4-D degradation using the bacteria *Arthrobacter* D9, *Pseudomonas* H1 and *Bacillus* H9 at pH 6 and temperature of 28°C.

#### High levels of NaCl, but not KNO_3_ inhibits the 2,4-D degradation

**Fig 3:**
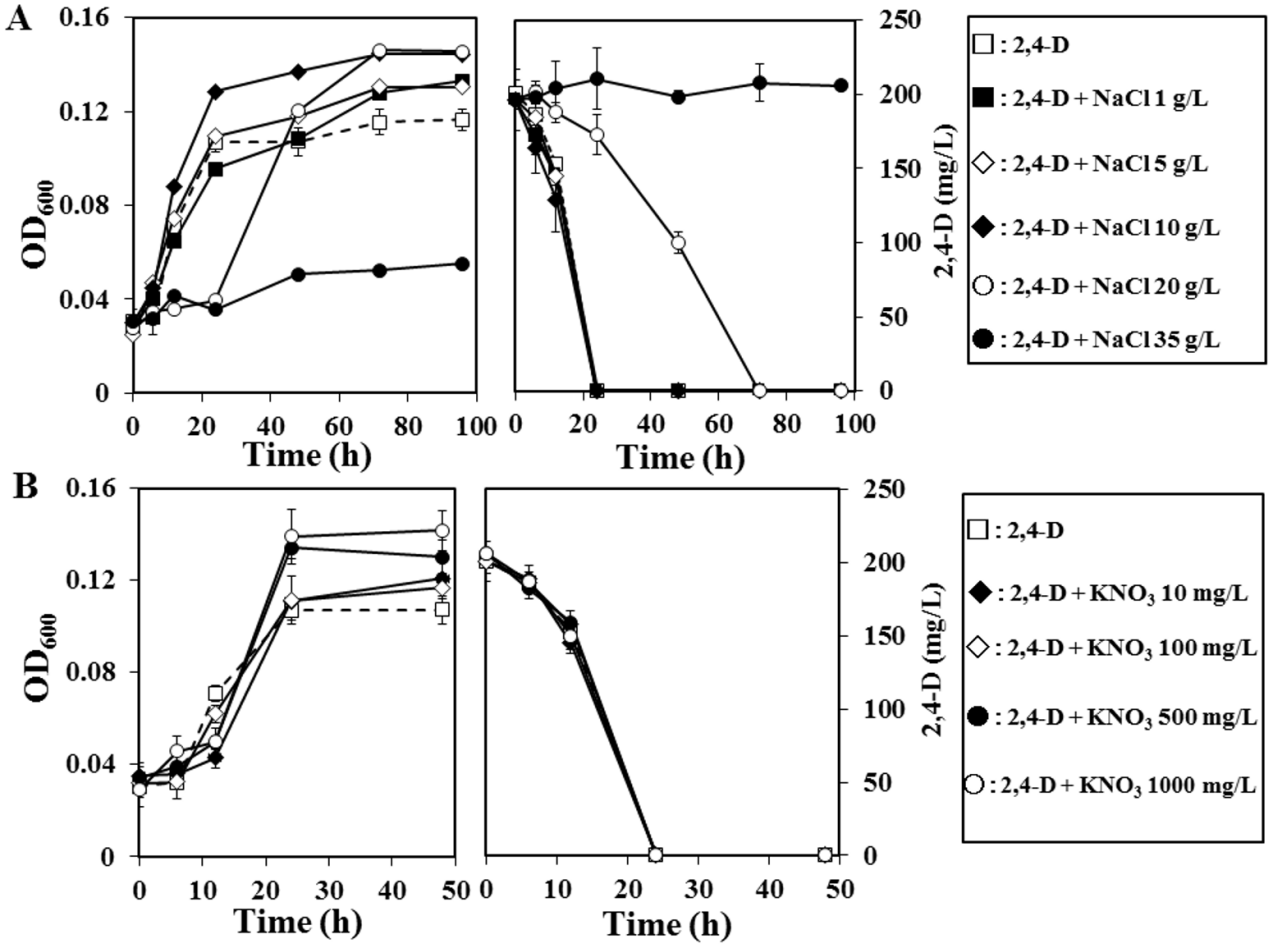
Growth curve and degradation of 2,4-D at 200 mg l^-1^ concentration by varying (A) NaCl (0.05 to 35 g l^-1^) and (B) KNO_3_ (0 to 1000 mg l^-1^) concentrations using *Cupriavidus* sp. CY-1.

The results of cell growth and 2,4-D degradation at different concentration of NaCl and KNO_3_ were shown in Fig 3. In the control (MS medium contains 0.05 g l^-1^ of NaCl), 2,4-D was completely degraded within 48 h (Fig 3A). NaCl up to 10 g l^-1^ did not showed any significant influence on the 2,4-D degradation efficiency of CY-1 strain, the results were almost equal with control (Fig 3A). But at 20 g l^-1^ of NaCl concentration the degradation of 2,4-D was delayed, CY-1 took 72 h for complete degradation of 2,4-D. However, at 35 g l^-1^ of NaCl concentration CY-1 was unable to degrade 2,4-D and also showed poor growth (Fig 3A). These results indicates that up to 20 g l^-1^ NaCl concentration, 2,4-D degradation efficiency of strain CY-1 was not affected. But at 35 g l^-1^ of NaCl concentration 2,4-D degradation efficiency of strain CY-1 was reduced. Contrary to this, 2,4-D degradation activity of strain CY-1 was not affected in the presence of KNO_3_ (up to 1000 mg l^-1^). Even at 1000 mg l^-1^ of KNO_3_, strain CY-1 proliferated well and degraded 2,4-D completely within 24 h (Fig 3B).

#### Chloride ions and 2,4-D metabolites determination

**Fig 4:**
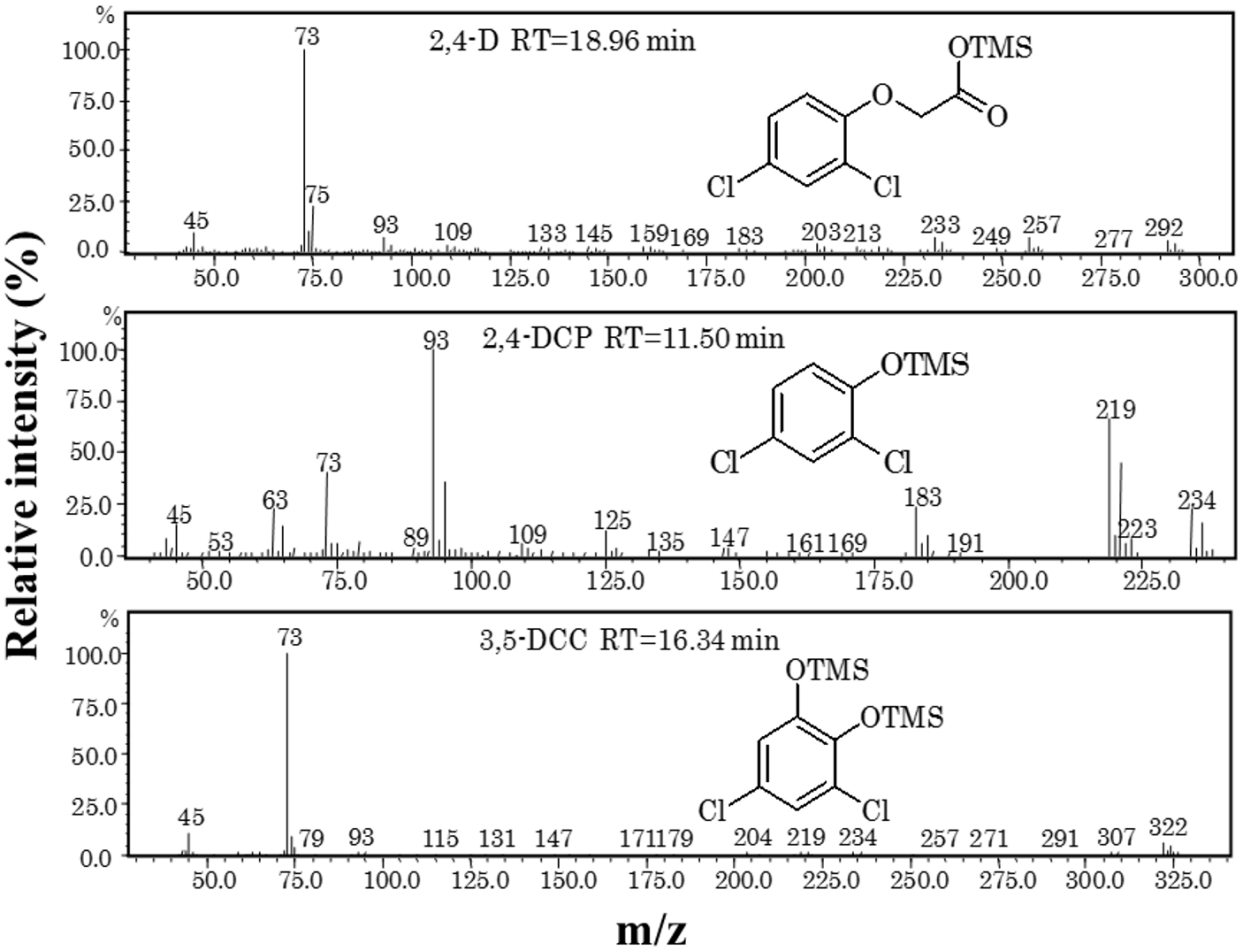
Mass spectra of TMS derivative of 2,4-D, 2,4-dichlorophenol (2,4-DCP) and 3,5-dichlorocatechol (3,5-DCC).

*Cupriavidus* sp. CY-1 completely dechlorinated 2,4-D within 24 h and the amount of chloride ions produced were stoichiometric with 2,4-D dechlorination (S3 Fig). The metabolites produced during 2,4-D degradation were analysed by GC-MS. Only three peaks from mass spectrographs were identified, which are corresponds to 2,4-D, 2,4-dichlorophenol and 3,5-dichlorocatechol (Fig 4). The maximum molecule ion at m/z = 292, m/z = 234 and m/z = 322 was presumed to be a TMS derivative of 2,4-D, 2,4-dichlorophenol and 3,5-dichlorocatechol respectively. The metabolite 3,5-dichlorocatechol was well oxidized by strain CY-1. Therefore, strain CY-1 could be very useful bacteria for effective bioremediation of 2,4-D-contaminated soil without release of toxic metabolites. Fukumori and Hausinger [35] also found similar types of metabolites during 2,4-D degradation using *Alcaligenes eutrophus*. However, other degradation intermediates such as 2,4-dichloro-*cis*,*cis*-muconate and *trans*-2-chlorodienelactone were not observed in the present study.

### Bio-augmented soil showed higher 2,4-D degradation

**Fig 5:**
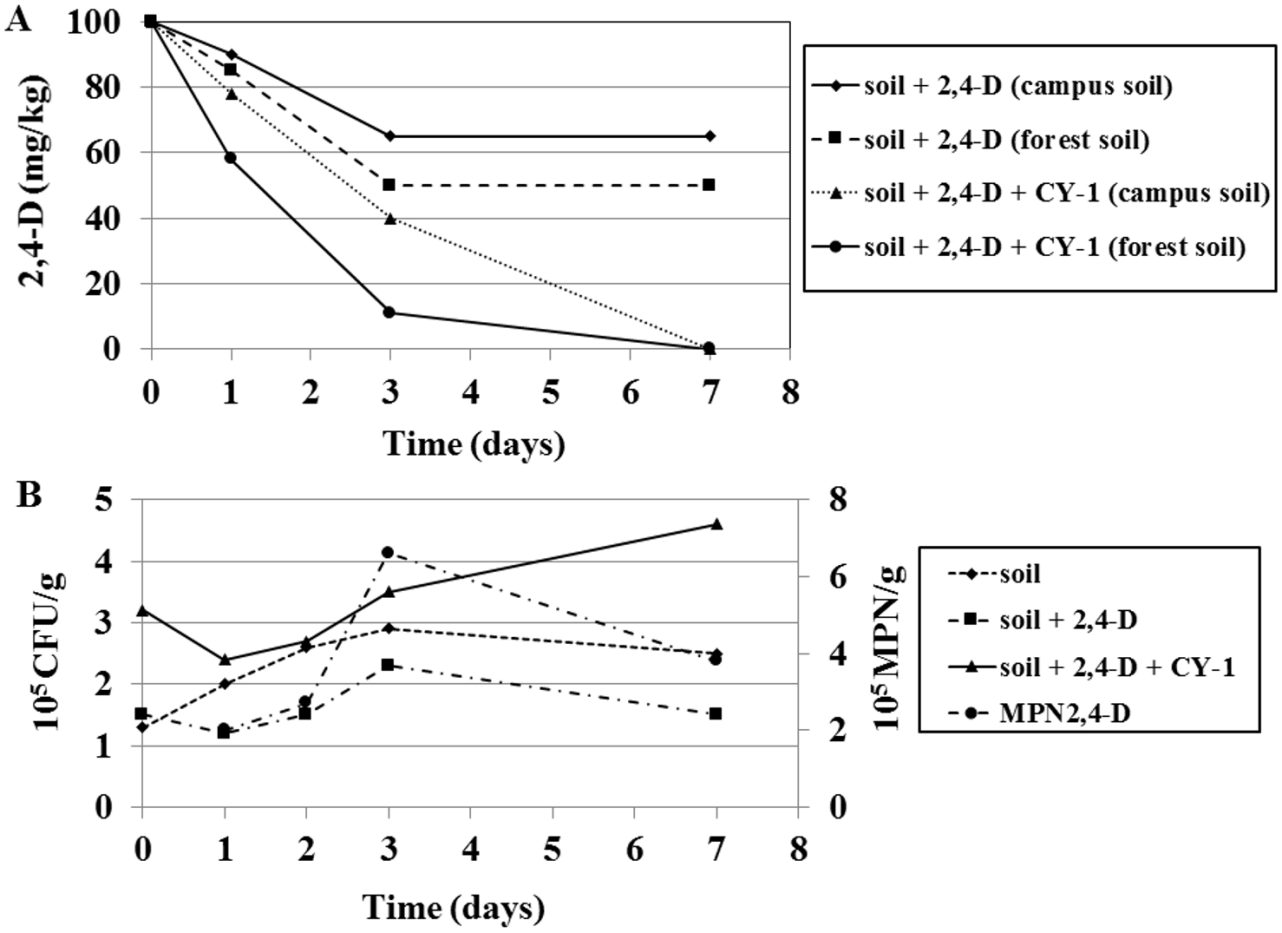
(A) Degradation of 2,4-D with MMIT campus soil and forest soil under non-bioaugmentation and bio-augmentation conditions. Soils were contaminated with 100 mg/kg of 2,4-D. For bio-augmentation experiments, 4 ml of CY-1 culture solution was inoculated into soils. Samples were collected at different time intervals and residual concentration of 2,4-D in the medium were analyzed by HPLC. (B) CFU and MPN of forest soil under non-bioaugmentation and bio-augmentation conditions.

For both non-bioaugmentation and bio-augmentation experiments soils were contaminated with 100 mg/kg of 2,4-D. Experiments were carried out for 7 days. Samples were collected at different time intervals *i*.*e*., 0 day, 1 day, 3 day and 7 day for HPLC analysis. Bio-augmented soils showed complete removal of 2,4-D within 7 days, but non-bioaugmented soils does not showed complete removal. Among non-bioaugmented soils, forest soil showed higher removal (50%) of 2,4-D than MMIT campus soil (35%) (Fig 5A). Among bio-augmented soils, forest soil showed fast removal (90%) of 2,4-D than MMIT campus soil (60%) within 3 days (Fig 5A). Existing reports denoted that bio-augmentation of *Cupriavidus* group of bacteria in to soils enhanced the degradation of toxic compounds. Newby et al. [36] conducted a pilot field study to assess the impact of bio-augmentation with the *Ralstonia eutropha* JMP134 for 2,4-D degradation at the concentration of 500 mg/kg soil. Bio-augmentation with *R*. *eutropha* JMP134 significantly increased 2,4-D degradation compared to control bioreactors. Approximately 98% of the 2,4-D was degraded within 21 days. In our study forest soil bio-augmented with CY-1 took 7 days for complete removal of 2,4-D at the concentration of 100 mg/kg soil. Roane et al [34] examined the potential of cadmium-resistant microorganisms to reduce soluble cadmium levels to enhance degradation of 2,4-D under conditions of co-contamination. In a pilot field study conducted with 18.92 liters soil bioreactors, the dual-bio-augmentation strategy was evaluated for 2,4-D degradation at the concentration of 500 mg/kg soil. They reported that the cadmium resistant isolate *Pseudomonas* strain H1 enhanced degradation of 2,4-D in reactors inoculated with *R*. *eutropha* JMP134 in the presence of 60 mg of cadmium per kg soil and overall dual bio-augmentation appears to be a viable approach in the remediation of co-contaminated soils. Chen et al. [37] developed symbiotic combination of an indigenous metal resistant rhizobial strain, *Cupriavidus taiwanensis* TJ208, and its host plant *Mimosa pudica* for the removal of heavy-metal pollutants. They reported, with *C*. *taiwanensis* TJ208 nodules the *M*. *pudica* plant exhibited a 71%, 81%, and 33% enhancement in metal adsorption efficiency for Pb, Cu, and Cd respectively. They finally concluded that bio-augmented plants showed higher removal efficiency than free-living *C*. *taiwanensis* TJ208.

#### Presence of heterotrophic and 2,4-D degrading bacteria

Aerobic heterotrophic bacteria present in the forest soil were identified by CFU. CFU count clearly shows the number of viable cells present in forest soil, forest soil contaminated with 2,4-D, and CY-1 bio-augmented forest soil contaminated with 2,4-D (Fig 5B). Among the three samples, CY-1 bio-augmented samples showed higher number of viable cells, followed by forest soil. Forest soil contaminated with 2,4-D showed lower number of viable cells, this may be due to the toxic nature of 2,4-D on indigenous bacteria present in the forest soil. CY-1 bio-augmented soil showed continuous increment of viable cells from 0 to 7 day, but forest soil and soil contaminated with 2,4-D showed increment of viable cells from 0 to 3 days, after that viable cell count was decreased. It was very well correlated with HPLC analysis. CY-1 bio-augmented soil showed 100% removal of 2,4-D, but non-bioaugmented soil showed only 50% removal.

2,4-D-degrading bacteria present in the forest soil were identified by MPN. Fig 5B clearly shows the increment of 2,4-D-degrading bacteria in MPN_2,4-D_ analysis. MPN_2,4-D_ was increased during 3 days of incubation and was decreased there after (Fig 5B). It was very well correlated with HPLC and DGGE analysis. In HPLC analysis, 90% of 2,4-D degradation was observed within 3 days and also high number of bands were observed in DGGE analysis at 3 day compared to 0 day and 1 day. These results demonstrated that bio-augmentation of CY-1 in to 2,4-D contaminated soils speed up the bioremediation process and also enhances the degradation efficiency of soil bacteria. On the other hand, when we tested the MPN_2,4-D_ of 2,4-D contaminated soil without bio-augmentation of CY-1, MPN_2,4-D_ showed decrement (data not shown) and only 50% of 2,4-D was removed from the soil within 7 days. Therefore, it could be assumed that indigenous bacteria present in the soil co-metabolized with 2,4-D using nutrients existed in the 2,4-D contaminated soil.

#### Dominance of *Firmicutes* in 2,4-D contaminated soil

**Fig 6:**
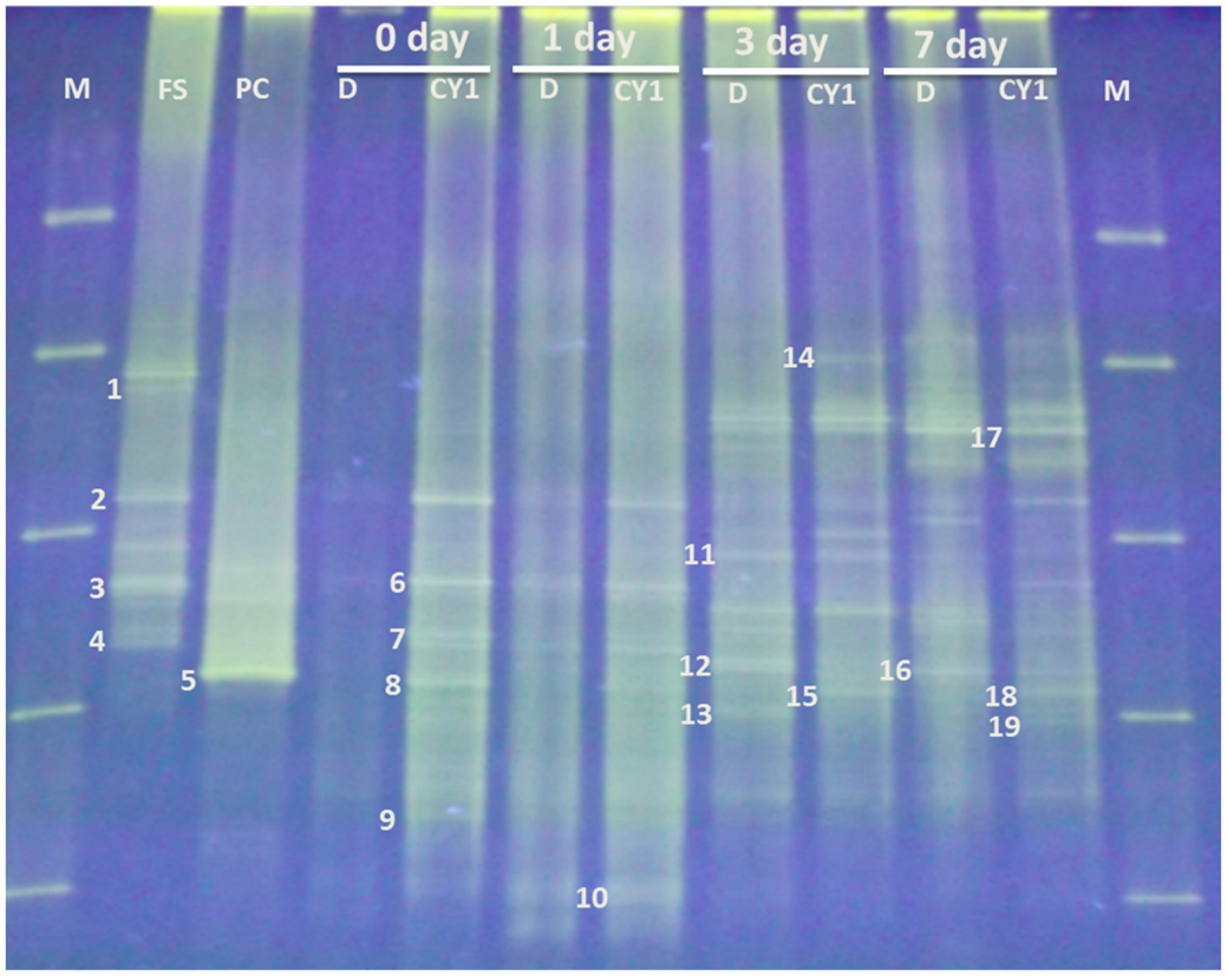
DGGE profile of 16S rDNA of bacterial community present in the forest soil. M: Marker; FS: Forest soil; PC: Pure culture; D: 2,4-D contaminated soil; CY1: 2,4-D contaminated soil bio-augmented with CY-1 strain.

DGGE outlines produced structural composition of bacteria present in the forest soil during 0, 1, 3 and 7 days of experiment (Fig 6). Among all the bands, 19 dominant bands were observed, which were phylogenetically related to class *Firmicutes* (53%) followed by *Bacteroidia* (27%), *β-proteobacteria* (10%), *α-proteobacteria* (5%) and *Enterobacteriales* (5%) (Table 2). *Firmicutes* and its members *Bacillus* were found to be predominant among all the classes. *Bacillus* are gram positive, motile, aerobic bacteria. These are the leading 2,4-D degraders, during the diversity studies the population was observed to be dominant on 3 day and 7 day, but low on 0 day. Many studies reported the capacity of *Bacillus* sp. to degrade 2,4-D. Han et al. reported nine *Bacillus* sp. strains isolated from soil degraded 50–81% of the 2,4-D at 500 mg l^-1^ initial concentration [26]. After *Firmicutes*, uncultured bacteria belongs to *Bacteroidia* were found to be prevailing. *Clostridium* sp. and *Enterobacter* sp. also found in the forest soil. Presence of these potential species of *Bacillus*, *Clostridium* and *Enterobacter* in the soil sustained the effective degradation of the 2,4-D.

**Table 2:** Bacteria identified in the forest soil based on DGGE analysis.

Band No	Closest relative	% Identity	Accession no	Phylogenetic affiliation
1	*Uncultured Bacillus* sp.	80	EU852238.1	*Firmicutes*
2	*Bacillus acidiceler*	98	KF475796.1	*Firmicutes*
3	*Uncultured bacterium*	93	EU466059.1	*Bacteroidia*
4	*Paenibacillus* sp.	86	KJ469906.1	*Firmicutes*
5	*Cupriavidus* sp.	99	AB604176.1	*β-proteobacteria*
6	*Bacillus* sp.	92	KC236718.1	*Firmicutes*
7	*Sulfobacillus* sp.	100	DQ673613.1	*Firmicutes*
8	*Cupriavidus* sp.	95	AB604176.1	*β-proteobacteria*
9	*Uncultured bacterium*	83	AB629174.1	*Bacteroidia*
10	*Uncultured bacterium*	97	KJ015967.1	*Bacteroidia*
11	*Uncultured Enterobacter* sp.	100	KJ526996.1	*Enterobacteriales*
12	*Bacillus altitudinis*	100	KM047502.1	*Firmicutes*
13	*Uncultured Clostridium* sp.	100	JX229148.1	*Firmicutes*
14	*Uncultured bacterium*	86	HQ323167.1	*Bacteroidia*
15	*Bacillus anthracis*	88	JQ271809.1	*Firmicutes*
16	*Hyphomicrobium* sp.	69	AF148858.2	*α-proteobacteria*
17	*Clostridium* sp.	99	JQ420049.1	*Firmicutes*
18	*Uncultured bacterium*	75	LN567904.1	*Bacteroidia*
19	*Bacillus cereus*	72	KC663436.1	*Firmicutes*

Both the incidence and concentration of the organisms were varied in co-ordination to the time intervals. Forest soil without 2,4-D addition showed dominant species of *Bacillus* and uncultured bacterium sp. (bands 1–4). After 2,4-D addition at 0 day very light bands and nonexistence of some bands were perceived. This indicates 2,4-D inhibited the bacterial growth at initial days due to its toxic nature. After 0 day, sharp increase in the number of 2,4-D degrading microbial populations with increase in time (during time intervals 1 day, 3 day and 7 day) were observed, which was well correlated with the observed 2,4-D degradation. *Enterobacter* sp. (band no. 11) are absent on 0 day and 1 day, but showed their presence on 3 day and 7 day due to the toxic nature of 2,4-D at initial days. As shown in Fig 6, substantial changes of band profiles on 0 day and 1 day incubation, were not observed. In divergence, the number of bands were increased after 3 days and 7 days of incubation under both bio-augmented and non-bioaugmented conditions. As a result, it was proved that indigenous bacteria present in the two conditions were activated owing to removal of 2,4-D. In the case of non-bioaugmented soils, most of the 2,4-D degradation was observed on day 3 (Fig 5A) and also good number of bands were observed. However, on day 7 there is no progress in 2,4-D degradation and some bands were no longer being visualized. It seemed to be owing to depletion of nutrients in soils. This result indicate that the microbial community was decreased and indigenous bacteria present in soil were 2,4-D co-metabolizing bacteria, not 2,4-D utilizing bacteria. When compared with the results of CFU, microbial community seemed to be in associated with viable cell counts.

In the case of bio-augmented soil, microbial community seemed to be in associated with CFU, because on day 3 the more number of viable cells were observed. As shown in Fig 6, on day 7 also variable microbial communities were observed. Survivability of strain CY-1 was also identified by loading the pure strain of CY-1 in to DGGE gel. Pure strain CY-1 showed clear band in DGGE gel (band no. 5), the band at same position in DGGE gel was appeared in bio-augmentation experiment in all time intervals (0, 1, 3, 7 day). In sequencing analysis also band number 5 showed 99% similarity with *Cupriavidus* sp. So it was confirmed that survivability of *Cupriavidus* sp. CY-1 (band no. 8) at 0 day. At same position bands were observed at 1 day, 3 day and 7 day. This result clearly denoted that strain CY-1 utilized 2,4-D, as a result toxic 2,4-D was removed from the soil, which may promote the growth of other indigenous microorganisms. Sustained growth of CY-1 has been observed after complete depletion of 2,4-D on day 7. These results indicated that strain CY-1 effectively degraded 2,4-D without disturbing the ecosystem of indigenous microorganisms.

#### Survivability of strain CY-1 in 2,4-D contaminated soil

**Fig 7:**
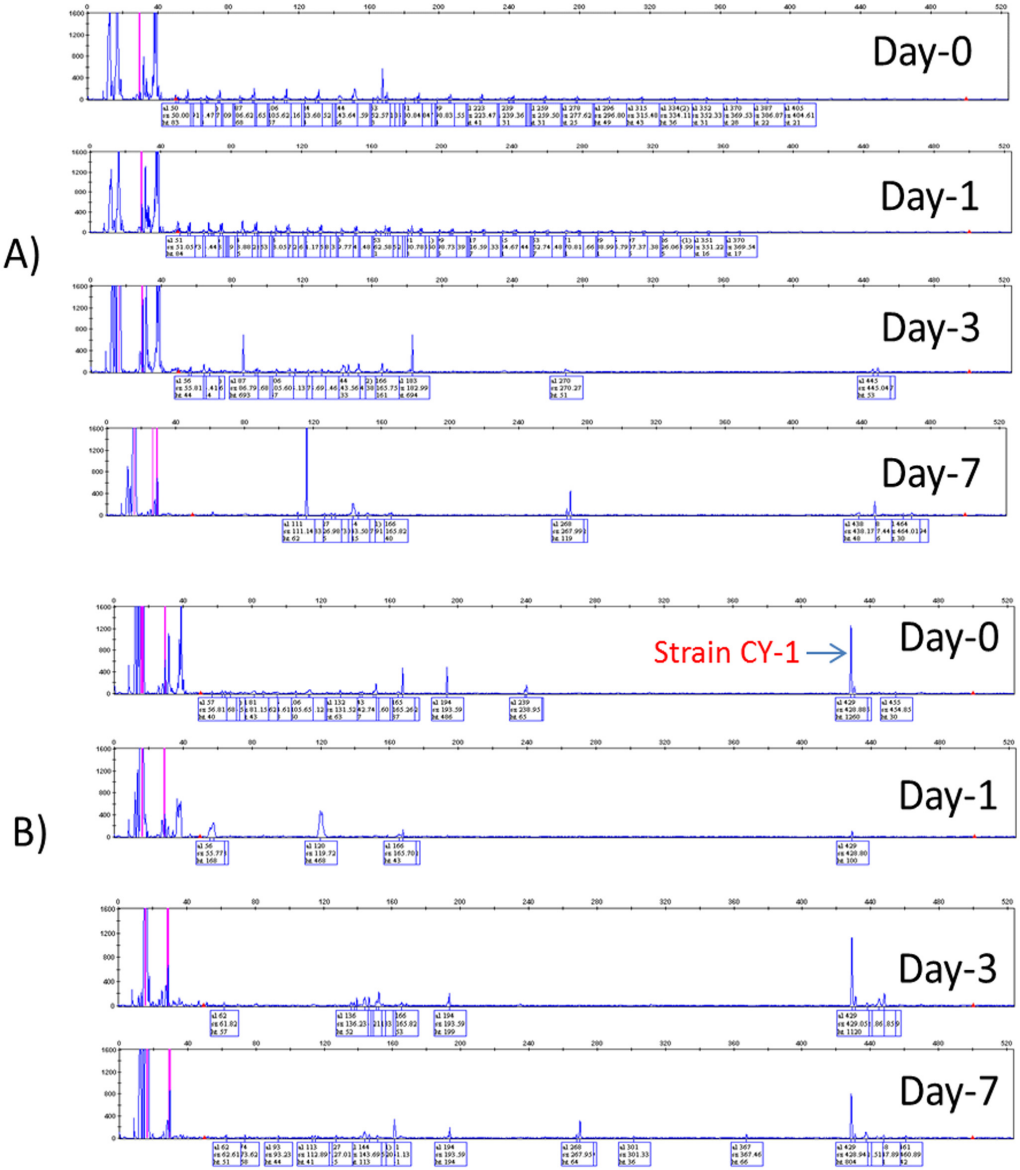
Electropherograms of T-RFLP analysis of (A) non-bioaugmentation (2,4-D + soil sample); (B) bio-augmentation (2,4-D + soil + CY-1 sample) conditions using forest soil samples.

T-RFLP analysis was done for both bio-augmented and non-bioaugmented soil samples to analyze the survivability of CY-1 strain. In T-RFLP analysis vertical and horizontal axis represents peak intensity and base pairs respectively (Fig 7). The peak observed at 492 bp in bio-augmented soil samples in all time intervals were derived from strain CY-1. This result was very well correlated with the viable cell count evaluation, *i*.*e*., on day 1 low peak intensity was observed and in viable cell count also low number of bacteria were observed. On day 3 and day 7 peak intensity was increased in accordance with increase of viable cell count. As shown in Fig 7, there is no significant change of soil microcosm under both bio-augmented and non-bioaugmented conditions. Manzano et al. [3] reported similar results that the introduction of *Cupriavidus* sp. JMP134 into native soil microcosm exposed to high levels of 2,4-D did not produce detectable changes in the structure of the bacterial community, as judged by 16S rRNA gene T-RFLP analysis. On the other hand, more accurate/or visible change of soil microcosm was observed with DGGE analysis rather than T-RFLP. Therefore, it could be concluded that the combination of two analysis provided a more accurate assessment of microbial diversity associated with 2,4-D degradation in soil.

Although, several authors [38–43] compared the potentiality of DGGE and T-RFLP to unravel the diversity of bacterial communities from complex environmental samples, to the best of our knowledge, this is the first study reporting on the potential of the two methods to evaluate the diversity of bacterial communities during the bioremediation of herbicide. In this study, traditional cultivation technique, MPN_2,4-D_ also given valuable information. Utilization of 2,4-D by CY-1 was confirmed and at the same time it was analyze that bacteria present in soil were co-metabolic 2,4-D degrading bacteria. These results were also supported by the viable cell count and DGGE result. In this study, most results obtained using viable cell counts and MPN_2,4-D_ determination was coincident with band profiles obtained using DGGE. In addition, it was realized that those culture dependent and independent methods provide an detailed explanation of the leading microbial inhabitants linked with 2,4-D degradation in soil. Eventually, it could be concluded that the combination of culture dependent and independent methods were useful to understand the degradation mechanism as well as feasibility of strain introduced into contaminated soil.

## Conclusions

*Cupriavidus* sp. CY-1 was isolated from forest soil sample with no history of herbicide application. The strain CY-1 showed good growth and completely degraded 2,4-D at 500 mg l^-1^ concentration within 72 h. The amount of chloride ions produced during the reaction was stoichiometric with 2,4-D dechlorination. CY-1 growth and degradation of 2,4-D were not affected by KNO_3_, but reduced at 35 g l^-1^ of NaCl. In bio-augmentation experiments, strain CY-1 completely degraded 2,4-D present in the soil. Combination of culture dependent and independent methods provided more accurate and complementing assessment to understand the microbial populations and degradation mechanism associated with 2,4-D degradation in soil. All these results showed that *Cupriavidus* genus is a major degrader of 2,4-D and the isolated strain CY-1 is potential bacteria for the clean-up of 2,4-D-contaminated soils.

## Supporting Information

S1 Fig A neighbor-joining tree constructed using Mega 6.0 showing the phylogenetic relationship of 16S rDNA sequences of isolated strain *Cupriavidus* sp. CY-1 from closely related sequences from GenBank. Accession numbers at the GenBank of National Center for Biotechnology Information (NCBI) are shown in parenthesis.
(PDF)

S2 Fig Effect of (A) pH and (B) temperature on 2,4-D degradation. To evaluate the effect of pH and temperature on 2,4-D degradation, the enzyme extracts were prepared with ultrapure water, phosphate buffer, or Tris-HCl buffer.
(PDF)

S3 Fig Release of chloride ions during 2,4-D degradation. Chloride ions were measured by using Dionex ICS-1000 Ion chromatography.
(PDF)

S1 Table Conventional tests used for characterization of isolated strain CY-1. (PDF)
