# Supplementary material for: Bio-Augmentation of Cupriavidus sp. CY-1 into 2,4-D Contaminated Soil: Microbial Community Analysis by Culture Dependent and Independent Techniques
Source: PLoS One. 2015 Dec 28;10(12):e0145057. doi: 10.1371/journal.pone.0145057 (PMC4699198; doi:10.1371/journal.pone.0145057)
Supplement: S1 Fig — Accession numbers at the GenBank of National Center for Biotechnology Information (NCBI) are shown in parenthesis. (PDF) [file pone.0145057.s001.pdf]

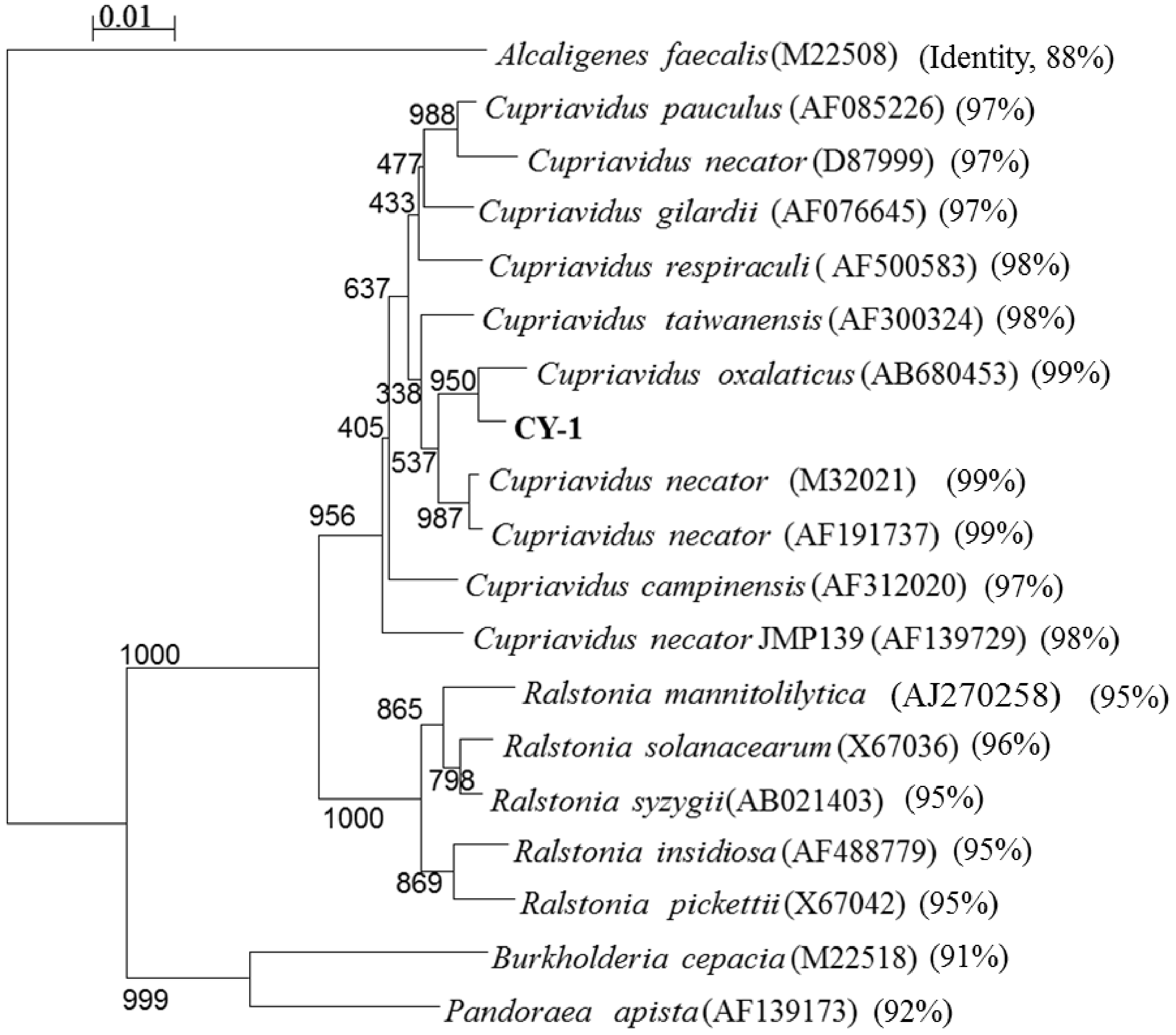

S1 Fig. A neighbor-joining tree constructed using Mega 6.0 showing the phylogenetic relationship of 16S rDNA sequences of isolated strain *Cupriavidus* sp. CY-1 from closely related sequences from GenBank. Accession numbers at the GenBank of National Center for Biotechnology Information (NCBI) are shown in parenthesis.
