# Supplementary material for: Bio-Augmentation of Cupriavidus sp. CY-1 into 2,4-D Contaminated Soil: Microbial Community Analysis by Culture Dependent and Independent Techniques
Source: PLoS One. 2015 Dec 28;10(12):e0145057. doi: 10.1371/journal.pone.0145057 (PMC4699198; doi:10.1371/journal.pone.0145057)
Supplement: S2 Fig — To evaluate the effect of pH and temperature on 2,4-D degradation, the enzyme extracts were prepared with ultrapure water, phosphate buffer, or Tris-HCl buffer. (PDF) [file pone.0145057.s002.pdf]

A

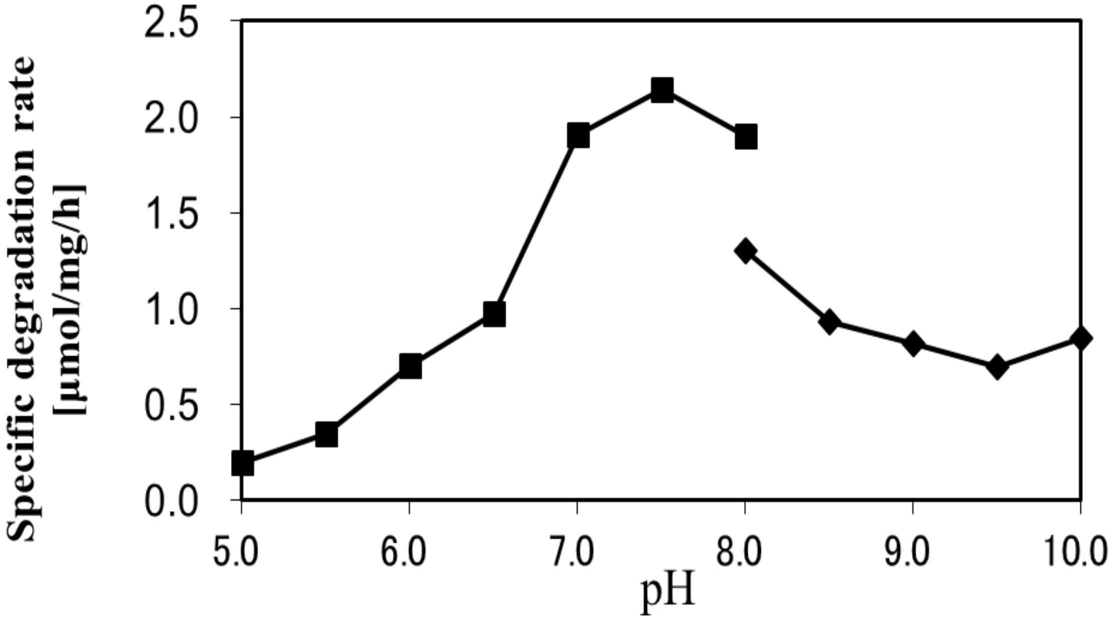

B

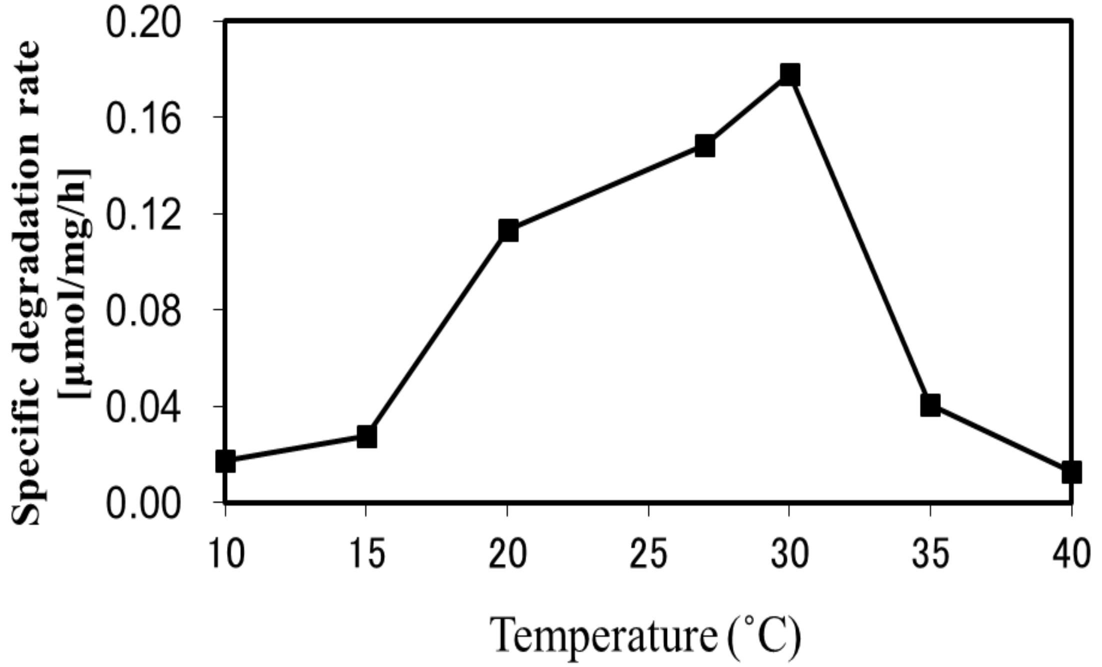

S2 Fig. Effect of (A) pH and (B) temperature on 2,4-D degradation. To evaluate the effect of pH and temperature on 2,4-D degradation, the enzyme extracts were prepared with ultrapure water, phosphate buffer, or Tris-HCl buffer.
