# Supplementary material for: Bio-Augmentation of Cupriavidus sp. CY-1 into 2,4-D Contaminated Soil: Microbial Community Analysis by Culture Dependent and Independent Techniques
Source: PLoS One. 2015 Dec 28;10(12):e0145057. doi: 10.1371/journal.pone.0145057 (PMC4699198; doi:10.1371/journal.pone.0145057)
Supplement: S3 Fig — Chloride ions were measured by using Dionex ICS-1000 Ion chromatography. (PDF) [file pone.0145057.s003.pdf]

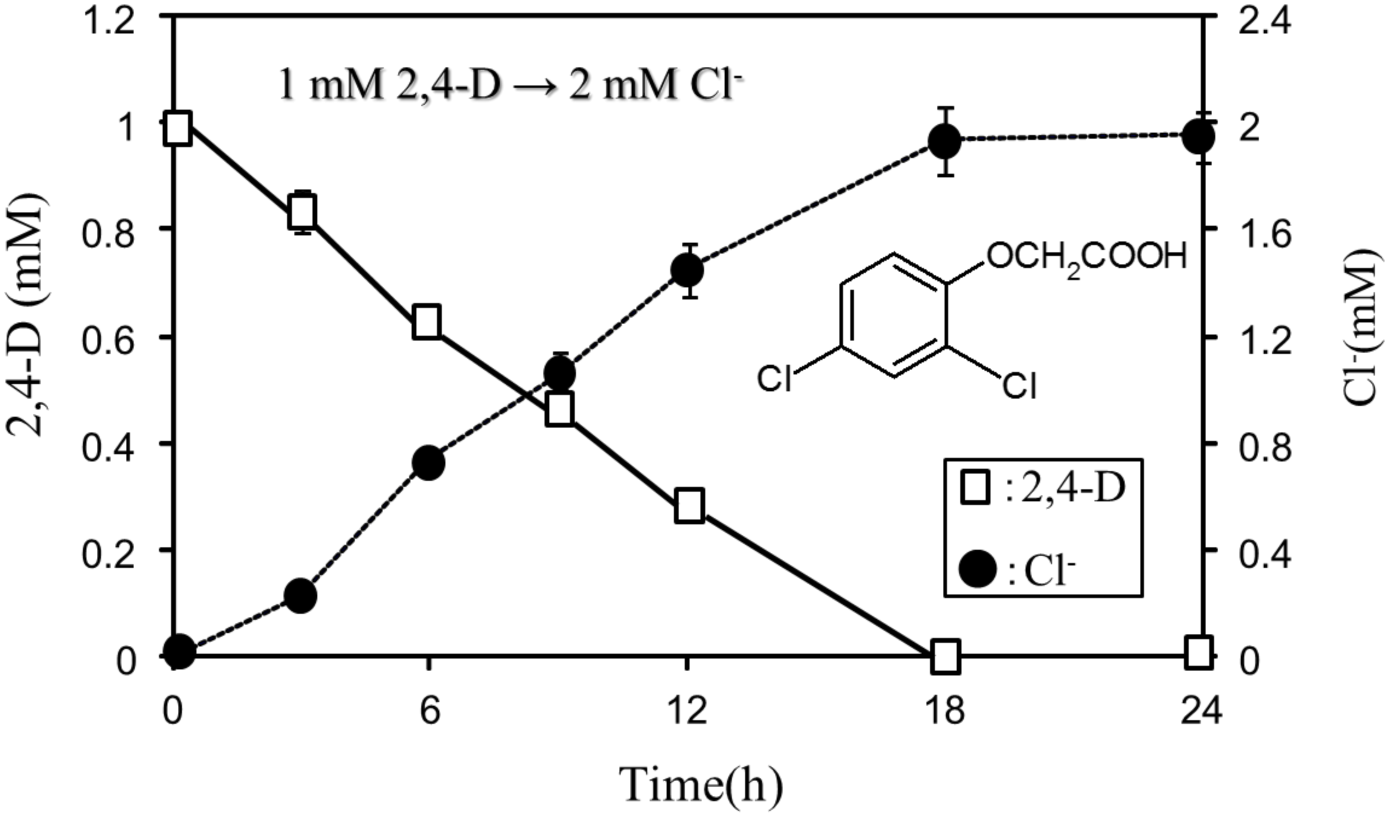

S3 Fig. Release of chloride ions during 2,4-D degradation. Chloride ions were measured by using Dionex ICS-1000 Ion chromatography.
