# Supplementary material for: Bio-Augmentation of Cupriavidus sp. CY-1 into 2,4-D Contaminated Soil: Microbial Community Analysis by Culture Dependent and Independent Techniques
Source: PLoS One. 2015 Dec 28;10(12):e0145057. doi: 10.1371/journal.pone.0145057 (PMC4699198; doi:10.1371/journal.pone.0145057)
Supplement: S1 Table — (PDF) [file pone.0145057.s004.pdf]

S1 Table: Conventional tests used for characterization of isolated strain CY-1.

**Physiological & biochemical tests**	**Result**
Shape	Rods
Gram reaction	Gram-negative
Motility	+
Spores	-
Growth in air	+
Anaerobic growth	-
Growth in 10% NaCl	-
Cytochrome oxidase	+
β-galactosidase acticity	-
Glucose fermentation	-
D-glucose utilization	-
Arabinose utilization	-
Mannose utilization	-
Mannitol utilization	-
N-acetylglucosamine utilization	-
Capric acid utilization	+
Maltose utilization	-
Adipic acid utilization	-
Gluconate potassium utilization	+
Malate utilization	+
Phenylacetic acid utilization	+
Citrate utilization	+
Esculin hydrolysis	-
Gelatin hydrolysis	-
Denitrogenation activity	-
Indole production	-
Urease test	+
Nitrate reduction	+ (fast)

+ Positive; - negative
